# Comparative genomics of methicillin-resistant *Staphylococcus aureus* ST239: distinct geographical variants in Beijing and Hong Kong

**DOI:** 10.1186/1471-2164-15-529

**Published:** 2014-06-26

**Authors:** Zheng Wang, Haokui Zhou, Hui Wang, Hongbin Chen, K K Leung, Stephen Tsui, Margaret Ip

**Affiliations:** Department of Microbiology, The Chinese University of Hong Kong, The Prince of Wales Hospital, Ngan Shing Street, Shatin, Hong Kong; Department of Clinical Laboratory, Peking University People’s Hospital, Beijing, 100044 China; School of Biomedical Sciences, The Chinese University of Hong Kong, Shatin, Hong Kong

**Keywords:** MRSA, Beijing, Hong Kong, Genome, Orthologous gene groups, ST239

## Abstract

**Background:**

The ST239 lineage is a globally disseminated, multiply drug-resistant hospital-associated methicillin-resistant *Staphylococcus aureus* (HA-MRSA). We performed whole-genome sequencing of representative HA-MRSA isolates of the ST239 lineage from bacteremic patients in hospitals in Hong Kong (HK) and Beijing (BJ) and compared them with three published complete genomes of ST239, namely T0131, TW20 and JKD6008. Orthologous gene group (OGG) analyses of the Hong Kong and Beijing cluster strains were also undertaken.

**Results:**

Homology analysis, based on highest-percentage nucleotide identity, indicated that HK isolates were closely related to TW20, whereas BJ isolates were more closely related to T0131 from Tianjin. Phylogenetic analysis, incorporating a total of 30 isolates from different continents, revealed that strains from HK clustered with TW20 into the ‘Asian clade’, whereas BJ isolates and T0131 clustered closely with strains of the ‘Turkish clade’ from Eastern Europe. HK isolates contained the typical φSPβ-like prophage with the *SasX* gene similar to TW20. In contrast, BJ isolates contained a unique 15 kb PT1028-like prophage but lacked φSPβ-like and φSA1 prophages. Besides distinct mobile genetic elements (MGE) in the two clusters, OGG analyses and whole-genome alignment of these clusters highlighted differences in genes located in the core genome, including the identification of single nucleotide deletions in several genes, resulting in frameshift mutations and the subsequent predicted truncation of encoded proteins involved in metabolism and antimicrobial resistance.

**Conclusions:**

Comparative genomics, based on *de novo* assembly and deep sequencing of HK and BJ strains, revealed different origins of the ST239 lineage in northern and southern China and identified differences between the two clades at single nucleotide polymorphism (SNP), core gene and MGE levels. The results suggest that ST239 strains isolated in Hong Kong since the 1990s belong to the Asian clade, present mainly in southern Asia, whereas those that emerged in northern China were of a distinct origin, reflecting the complexity of dissemination and the dynamic evolution of this ST239 lineage.

**Electronic supplementary material:**

The online version of this article (doi:10.1186/1471-2164-15-529) contains supplementary material, which is available to authorized users.

## Background

The ST239 lineage of methicillin-resistant *Staphylococcus aureus* (MRSA) is one of the most widely disseminated hospital-associated MRSAs (HA-MRSA) [1], which has caused multiple epidemics around the world in recent decades. In China, as in most Asian countries, ST239-SCC*mec*III has been identified as the predominant clone, accounting for around 75% of observed HA-MRSA [2, 3]. In Hong Kong, ST239-SCC*mec*III was the most prevalent MRSA clone during the late 1980s and the 1990s [4, 5]. However, although it remains one of the most common clones in hospitals in Hong Kong, its prevalence amongst ST239 strains has decreased from the early 2000s [6]. Whole-genome sequencing in clinical microbiology has revolutionized our understanding of MRSA, in areas such as outbreak investigation [7, 8], evolutionary and phylogeographic distribution and in recombination studies [9, 10]. Harris *et al.* [9] described the comparative genomics, by the reads-mapping method, of globally collected ST239 strains using the TW20 genome as a reference. This research group demonstrated the global geographic distribution of the ST239 lineage, indicating intercontinental transmission, based on core-genome single nucleotide polymorphisms (SNPs) [9]. The ST239 lineage consisted of more than five MRSA clades; these reflect the various continental origins, such as North and South America, Australia and Europe, whereas the TW20, Chinese and Thai isolates formed into the single ‘Asian clade’ [9]. Ramirez *et al.* supplemented these data with further isolates, confirmed the strong geographical clustering and identified recombination rates that varied between phylogeographic sub-groups [10]. Marked divergence was noted between the European ST239 strains. Prophages, as one form of mobile genetic element (MGE), play an important role in horizontal gene transfer and the bacterial evolution of MRSA. The φSPβ-like prophage is thought to be an important characteristic of the ST239 ‘Asian clade’ [9, 10], as it possesses the *Sas*X gene, a crucial pathogenicity determinant in the spread of ST239 [1].

In this study, we performed whole-genome sequencing of four clinical isolates of MRSA that were representative of HA-MRSA ST239 isolated in Hong Kong and Beijing during different time periods. We investigated the genomic diversity and evolutionary origins of these four isolates, by comparing their genomes with those of three previously published ST239 isolates and the publicly available ST239 sequence that represents strains from different continents. In addition, orthologous gene group (OGG) analyses from annotations and whole-genome alignments of the Hong Kong and Beijing strains were examined to highlight differences at the protein group level in the core and non-core regions that distinguish and characterize these strains geographically.

## Results

### The phylogenetic analysis of ST239 clones

**Figure 1:**
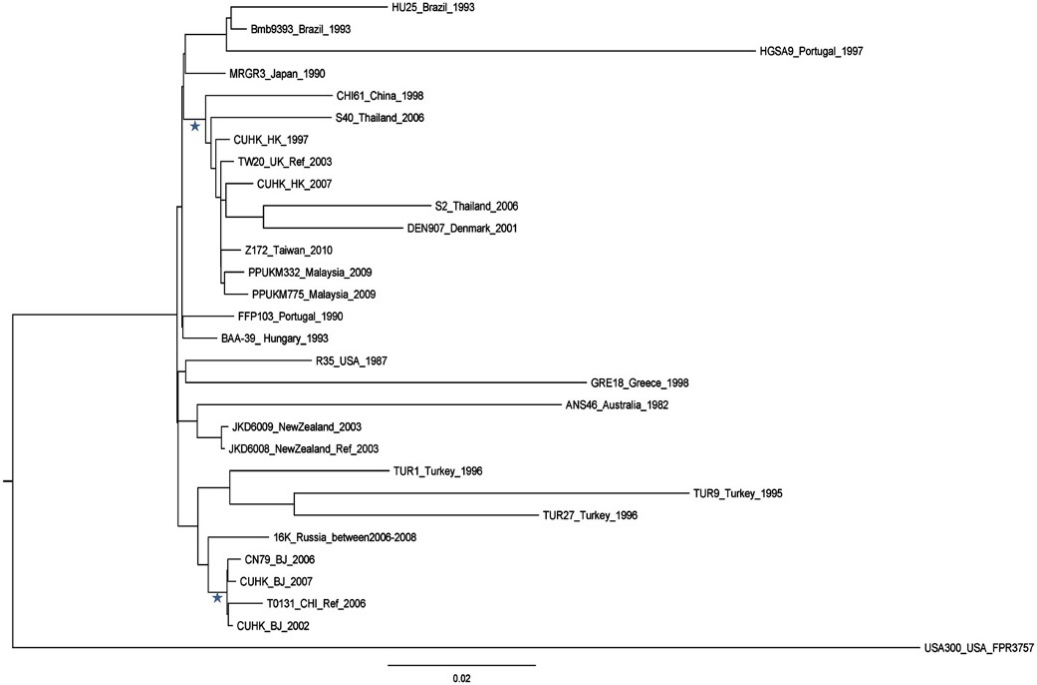
**Maximum likelihood phylogenetic tree of ST239.** The phylogeny was based on the SNPs of the core genomes. The tree was rooted by using MRSA FPR3757 USA300 as an outgroup. The stars represent 100% bootstrap support.

The molecular types of the strains are summarized in Table 1. The relationship of the HK and BJ strains, relative to other representative global ST239 isolates, is shown in the maximum likelihood phylogenetic tree in Figure 1. A total of 2767 core genome SNPs were identified among these 30 isolates. Distinct clustering between the HK (HK97, HK07) and BJ (BJ02, BJ07) strains was obtained. The two HK strains showed a close relationship and they were clustered within the ‘Asian clade’ with TW20, previously reported strains from China (CHI62, 1998) and Thailand (S2, S40, 2006), and DEN907 from Denmark [9]. However, BJ strains formed a distinct cluster with reference T0131. The T0131 strain was recovered from an 87-year-old patient in Tianjin, northern China in 2006 [11]. This BJ cluster was closely related to the strains of the ‘Turkish clade’ (TUR1, TUR9 and TUR27) and the 'Russia variant' (16K) (Figure 1). This strongly suggested that these were of different origin to the ST239 strains in Hong Kong and Asia. The other ST239 strains showed consistent geographical clustering in concordance with previous studies by Harris *et al.* [9] and Santiago *et al.* [10].

**Table 1:** **Characteristics of MRSA isolates in study**

	Representative clinical isolates				ST239 MRSA reference strains		
	HK97	HK07	BJ02	BJ07	TW20_London_2003	T0131_Tianjin_2006	JKD6008_NewZealand_2003
CC	8	8	8	8	8	8	8
ST	239	239	239	239	239	239	239
spa	t037	t037	t030	t030	t037	t030	t037
SpA-repeats	15-12-16-2-25-17-24	15-12-16-2-25-17-24	15-12-16-2-24-24	15-12-16-2-24-24	15-12-16-2-25-17-24	15-12-16-2-24-24	15-12-16-2-25-17-24
SCCmec type	III	III	III	III	III	III	III

### Comparative genomics

**Figure 2:**
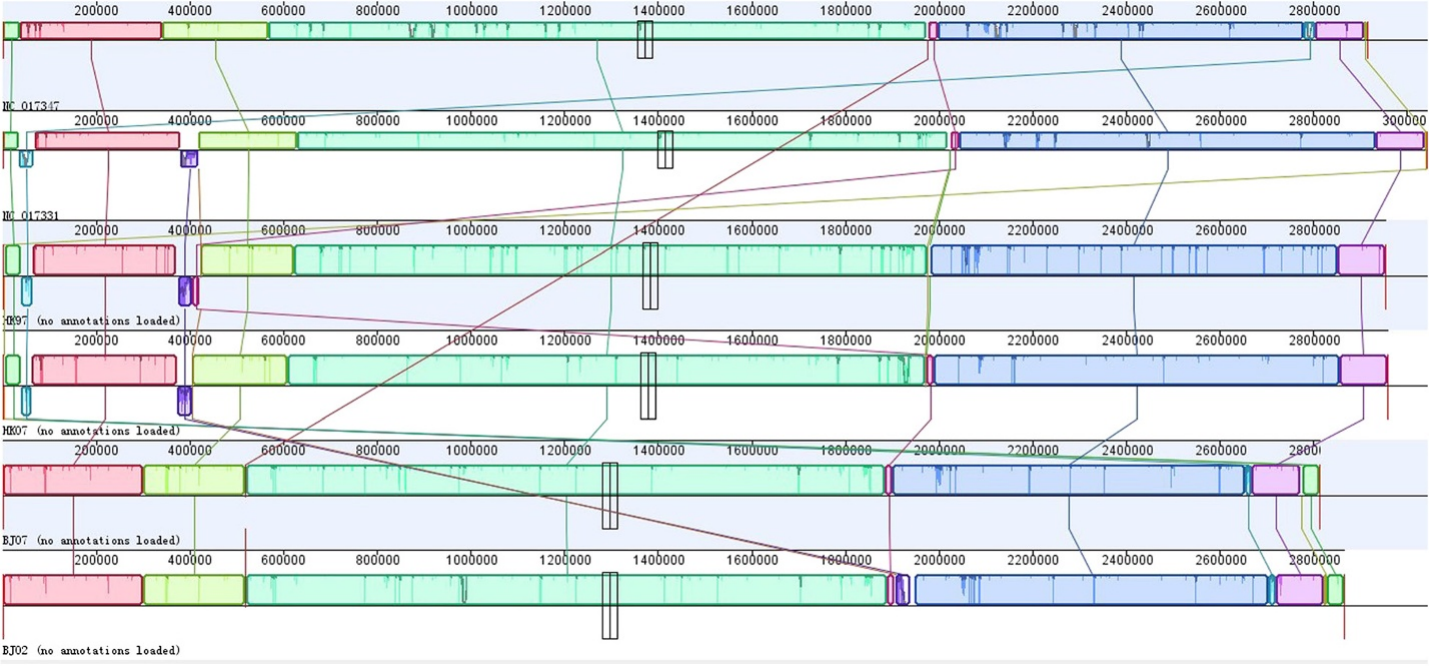
**Genome information for the Hong Kong cluster ST239 strains in comparison with the Beijing cluster.**

**Figure 3:**
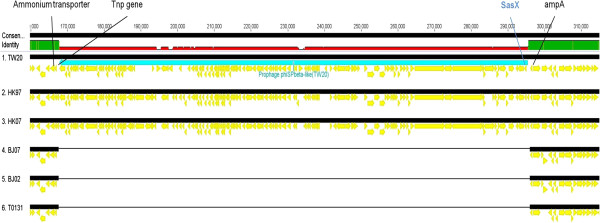
**The structure of prophage φSPβ-like(TW20) in HK vs. BJ genomes.**

The BJ02 and BJ07 genomes were estimated to be at least 2.8 Mb in size and the HK97 and HK07 genomes approximately 3.0 Mb (genome sequencing and contig details are shown in Table 2). Comparison with the three published complete genomes – T0131, TW20 and JKD6008 – revealed that the two BJ genomes have the highest average nucleotide similarity to T0131 (BJ02 99.998% and BJ07 99.997%), the lowest to TW20 (BJ02 99.931% and BJ07 99.934%) and the reverse held for the two HK strains (vs. T0131, HK07 99.958% and HK97 99.949%; vs. TW20, HK07 99.989% and HK97 99.992%). These results were consistent with those of the phylogenetic tree (Figure 1). The two BJ genome sequences and T0131 (described as the BJ cluster) were compared with the two HK genomes and TW20 (the HK cluster) by whole-genome alignment (Figure 2). The major difference between the two clusters was the presence or absence of specific MGEs, which also explained the difference in genome sizes. A φSPβ-like 127.2 kb (TW20) prophage was present in the HK97 and HK07 strains (Figure 3). In contrast, the whole φSPβ-like (TW20) prophage was absent in BJ02 and BJ07, instead replaced by a 1080 bp gene encoding a hypothetical protein between the *tnp* and *amp*A genes, similar to the reference genome, T0131. The φSPβ-like (TW20) prophage is considered to be a feature of the ST239 ‘Asian clade’ and it is therefore similarly detected in other ‘Asian clade’ ST239 strains (CHI61, S2, S40, TW20 and DEN907). These strains possess the *SasX* gene, which is located at the 3’ end of the φSPβ-like prophage and plays a key role in MRSA colonization, making it a crucial pathogenicity determinant in the spread of ST239 [1]. The *SasX* gene was absent in the BJ cluster.

**Table 2:** **Genome sequencing and contig assembly statistics**

Parameters	HK97	HK07	BJ02	BJ07
Total no. of reads	14273328	21087930	12905538	14503760
No. of assembled reads	13807870	20384885	12631772	14335583
No. of contigs	125	81	89	81
Total no. of bases	1284599520	1897913700	1161498420	1305338400
Average contig size (bases)	24068	37011	32465	35090
N50 contig size (bases)	53452	109354	79782	79076
Largest contig size (bases)	167992	206466	310965	258620
Coverage depth	424	629	400	457

**Figure 4:**
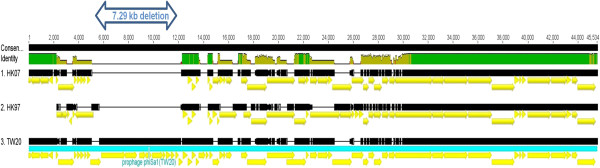
**The structure of prophage φSA1 in HK genomes.**

**Figure 5:**
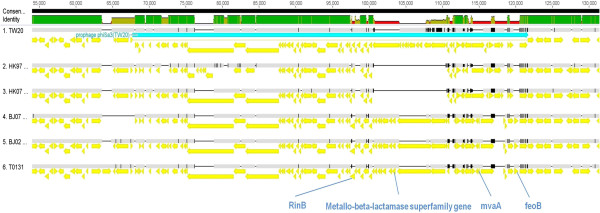
**The structure of prophage φSA3 in HK vs. BJ genomes.**

Another notable difference between the two clusters was the absence of the 43.4 kb prophage φSA1 in the BJ cluster. Both HK genomes contained a 7.29 kb deletion within φSA1 (Figure 4). It has previously been noted that the φSA1 region does not carry any known virulence factors [12]. Both clusters possessed the 44.7 kb prophage φSA3 but some differences were observed, especially in the region between the gene encoding the transcriptional activator RinB (SAT0131_02106), to the 3′ end (Figure 5). In this region, the BJ cluster contained the additional coding sequences (CDS): *mva*A, *φ*PVL hypothetical protein, metallo-beta-lactamase superfamily domain-containing protein and *feoB* Ss-1,3-N-acetylglucosaminyl transferase. The proteins encoded by these CDS are involved in antibiotic resistance, virulence and metabolism. BLAST analysis indicated that the *feo*B Ss-1,3-N-acetylglucosaminyl transferase gene was only identified previously in two Australian strains in the NCBI database: JKD6008 (MRSA ST239 clone) and JKD 6159 (MRSA ST93 clone). The metallo-beta-lactamase superfamily domain-containing protein gene has been found in a number of different phages and MRSA clones, such as *Staphylococcus* phages JS01, SP5 and phiNM3; and MRSA clones JKD6008 (MRSA ST239 clone), MRSA252 (MRSA ST36 clone), and 71193 (MRSA ST398 clone). TW20 also harboured a similar gene, but differed by 17 SNPs, and none of the HK strains harboured this gene. For the *mva*A *φ*PVL hypothetical protein gene, similar genes were found in JKD6008 (ST239 clone), JKD6159 (MRSA ST93 clone), MW2 (ST1 clone) and MSSA476 (MSSA ST1 clone). All six genomes possessed other virulence-associated genes, including the phospholipase C gene, staphylococcal complement inhibitor SCIN, staphylokinase and enterotoxin A [13].

**Figure 6:**
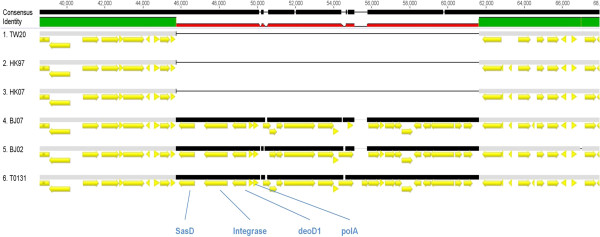
**The structure of prophage PT1028-like region in the BJ genomes.**

A noteworthy difference in the BJ cluster, absent in the HK strains, was a 15.5 kb region insertion in the gene encoding the 30S ribosomal protein S18, rpsR (SATW20_04350). BLAST and PHAST identified that this region shows high similarity with phage PT1028. This PT1028-like prophage contains important functional genes, such as *sas*D, integrase, *pol*A, *deo*D1, SaPI1 and others encoding pathogenicity island proteins (Figure 6).

The analysis also demonstrated other divergences at the MGE level. For example, the pathogenicity island SaPI1 (TW20), which contains enterotoxins K and Q, was absent in all of the BJ cluster strains. Similar to TW20, HK07 carried the SaPI1 island (100% homologous), but it was absent in HK97 (Detailed structures are illustrated in Additional file 1). An 18 kb insertion in T0131 in the chromosome downstream of the *rnr* putative ribonuclease gene, containing an exfoliative toxin A/B gene, was not present in the HK cluster. Both the BJ strains contained a 6.9 kb fragment of this 18 kb insertion region, but not the virulence genes (Additional file 2).

There are also unique features of individual strains. For example, PHAST indicated a specific 47.5 kb region in BJ02, which showed the greatest similarity with the prophage φNM1 (Newman strain). This φNM1-like prophage (BJ02) harbored two of the three virulence genes, *i.e.,* homologs of SAV0866 and SAV1978 [14], but not homologs of the SAV0862 gene. The detailed structure of this φNM1-like prophage (BJ02) is included in Additional file 3.

### OrthoMCL analysis

**Figure 7:**
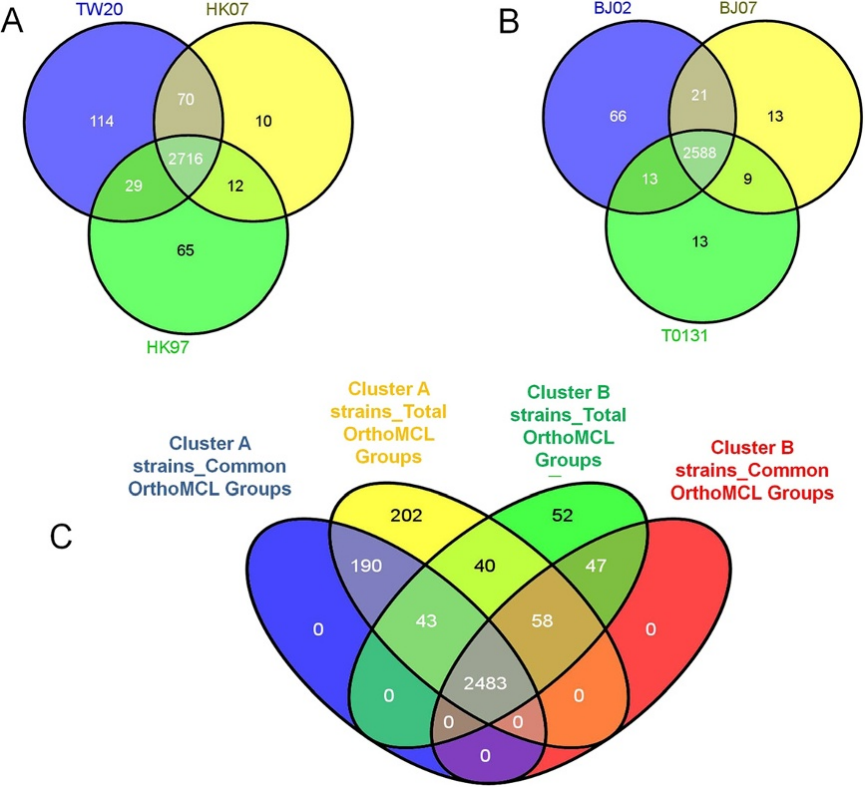
**Venn diagrams showing the number of orthologous groups in the two clusters of ST239 genomes. (A)** The common and unique orthologous groups among HK97, HK07 and reference TW20. **(B)** The common and unique orthologous groups among BJ02, BJ07 and reference T0131. **(C)** The relationship between Cluster A strains (HK97, HK07 and TW20) and Cluster B strains (BJ02, BJ07 and T0131). The overlapping blue ellipse shows 2,716 common orthologous groups present in all of Cluster A strains (*i.e.,* HK97, HK07 and TW20). The overlapping yellow ellipse shows the orthologous groups present in at least one of the Cluster A strains. The red ellipse shows 2,588 common orthologous groups present in all of the Cluster B strains (*i.e.,* BJ02, BJ07 and T0131). The green ellipse shows orthologous groups present in at least one of the Cluster B strains. The intersections of these four ellipses show the relationships of the identified orthologous groups in different genomes.

With annotation information and the orthoMCL algorithm, the families of orthologous gene groups were calculated by comparison with TW20 and T0131. The protein sequences of the latter reference genomes were derived from NCBI annotations. The results are illustrated in Venn diagrams (Figure 7). The HK cluster strains shared 2,716 common orthologous gene groups (OGGs) (Figure 7A). The BJ cluster strains shared 2,588 common OGGs (Figure 7B). BJ02 contained more unique OGGs not present in the other two genomes. Figure 7C shows that 2,483 common OGGs were shared by all six genomes.

Forty-seven OGGs were common in the BJ cluster but were not present in any of the HK cluster genomes. At least 27 of 47 OGGs were related to genomic islands (GIs). Sixteen orthologous groups were composed of hypothetical proteins and pseudogenes, whereas four OGGs consisted of genes orthologous to genes for the following representative proteins from T0131: histidine ammonia-lyase; myosin-cross-reactive streptococcal antigen homolog; methicillin-resistance-related protein *FmhC*; and multidrug resistance transporter B.

The details of these four OGGs are shown in Additional file 4. Genes in three of the groups (all except the multidrug resistance transporter B group) contained single nucleotide deletions, thus leading to a frameshift mutation and predicted truncation of their encoded proteins. The first group included the gene encoding histidine ammonia-lyase (SAT0131_00009) and its homologs in BJ02 and BJ07. The 504 aa hutH histidine ammonia-lyase protein (SAR0008) is present in MRSA strains such as MRSA252, JKD6008 and TW20 and plays an important role in histidine metabolism. In the BJ cluster strains, a ‘T’ deletion occurred at the 1,095th bp of the hutH gene (g.11505delT: NC_017347), causing a frameshift mutation that truncated the hutH into a 382aa length histidine ammonia-lyase protein (SAT0131_00008) and a 120aa protein SAT0131_00009. The putative conserved Lyase-I-like superfamily domain was detected in this 120 aa protein. Further functional analysis and experimental work are needed to determine whether this has any significant impact on histidine metabolism.

A frameshift mutation (g. 94707delT: NC_017347) was also detected in the BJ cluster strains of the 591 aa myosin-cross-reactive antigen (SAR0111) protein (SAT0131_00089), the representative protein in the second OGG. It is not known what the effects of this mutation might be.

The remaining two groups were related to antimicrobial resistance. Whole-genome alignment of the BJ cluster revealed an ‘A’ deletion (g.1281606delA: NC_017347) at the 826th bp position of the MW1131 gene (encoding the methicillin resistance-related protein FmhC). This frameshift mutation resulted in a truncated 281 aa protein SAT0131_01298 and a 128 aa length protein SAT0131_01299. The 414 aa length FmhC protein (MW1131) shares identity with FemA and FemB and is classified in the cell wall category [15]. Previously, it has been reported that insertional deactivation of FmhC had no effects on growth, antibiotic susceptibility or the lysostaphin resistance of *S. aureus* strains [16] and its function remains elusive.

In the fourth OGG, multidrug resistance protein B (SAT0131_01869) shared identity with the 393aa multidrug resistance transporter protein B (NWMN_1652), which is present in NEWMAN, COL, USA300_FPR3757, JKD6008 and TW20 MRSA strains. Whether specific local selective pressures by antimicrobials may have contributed to the changes described above, and how these alter antimicrobial resistance, remains to be determined.

The HK cluster genomes contained 190 OGGs that were not present in any of the BJ cluster genomes. Except for 18 groups, which consisted of hypothetical proteins and pseudo genes, all of the other 172 OGGs were associated with genomic islands (GIs). This may reflect the substantial impact of GIs on the evolution of the ‘Asian clade’ of ST239.

## Discussion

The ST239 lineage represents a globally disseminated multi-drug-resistant HA-MRSA. The geographical clustering of strains of the ST239 lineage has been confirmed, both at the continental and national levels [10], and the diversity of European isolates has been discussed in various studies [9, 10]. However, Asian isolates were thought to belong to a single, large ‘Asian clade’, as represented by Thai and Chinese isolates, although some diversity was detected within this clade [9]. Our data revealed geographical clustering – with a diversity of ST239s causing epidemics in hospitals in Beijing and Hong Kong during the late 1990s and the 2000s – and suggested different origins of the ST239s and the possibility of a more complex distribution in Asia. The HK isolates clustered within the traditional ‘Asian clade’ and showed a high similarity with TW20, and those present in Taiwan, Thailand and Malaysia. The distinct BJ cluster is more likely to represent the local establishment of an endemic, predominant clone for a number of years, at least from 2002 onwards. According to a recent national surveillance report of MRSA in China, ST239-III-t030s have the same molecular types and characteristics as CUHK_BJ02, and they remain the most predominant HA-MRSA clones in China, with an overall prevalence of 57.1% [2]. The BJ cluster, including the reference genome T0131, showed distinct features, based on their non-synonymous SNPs at the core genome, as well as differences at the MGE level. These observations collectively indicated the possibility that the BJ cluster ST239-III-t030s originated from a relatively recent common ancestor and was disseminated during this period in the ST239 lineage. The CN79 strain, isolated in 2006 and confirmed as a representative ST239 strain in Beijing [17], also clustered within the BJ cluster, and reaffirmed the homogeneity of this cluster. Interestingly, the ‘Russia variant’ 16K strain [18] also showed close relationship with the BJ cluster and ‘Turkish clade’, further illustrating the distinct geographical spread of ST239 in the north of China. Further studies are needed, on strains retrieved from older collections and across other parts of China, to estimate the origin and widespread nature of the BJ cluster of ST239.

The reads-mapping assembly method has inherent limitations for MGE detection and non-core genome analysis; this is because MGEs that are absent in the reference genome are not detectable. This disadvantage cannot be overcome, even by using a large number of reference genomes. Thus, in our study, we performed a *de novo* assembly and deep sequencing (more than 400X coverage depth in this study) of each strain. Moreover, three reference genomes were used in the ordering of contigs and the whole-genome alignment process. Recently, an ST239 Russian variant also showed an absence of the φSPβ-like (TW20) prophage [18] and this may suggest a potential evolutionary link. However, the unique PT1028-like prophage was not reported in the Russian isolate and the relationship of these strains remains elusive.

The isolates from Hong Kong are representative of the prevalent ST239 clone during the last two decades. ST239 has been the predominant HA-MRSA clone since 1988 and the strains were characterized as multidrug-resistant and prevalent in various hospitals in Hong Kong [4–6]. They are closely related to TW20 of the ‘Asian clade’ and possessed the φSPβ-like prophage and were *SasX-*gene-positive, and they further demonstrated the epidemic wave and dissemination of *sasX*-positive ST239 HA-MRSA in Hong Kong and southern China. The results of OrthoMCL analysis supported the phylogenetic tree at the protein level. The specific OGGs that were present only in the HK or BJ cluster strains provided further evidence that the horizontal gene transfer of GIs played an important role in ST239 family evolution and geographical clustering. Comparative genomics revealed the common differences between the two clades of ST239 HA-MRSA at SNP, gene and MGE levels. The availability of next generation sequencing on a wider scale will further enhance our understanding of the dynamic evolutionary process in the transmission and spread of globally disseminated multidrug-resistant MRSA.

## Conclusions

In summary, comparative genomics, based on *de novo* assembly and deep sequencing, revealed the different origins of the ST239 lineage in northern and southern China and pointed out the common differences between the two ST239 HA-MRSA clades at SNP, gene and protein levels. Besides distinct GIs, which were responsible for the major differences in the two clusters, orthoMCL analyses and whole-genome alignment of the HK and BJ clusters highlighted differences in genes located in the core genome. Single nucleotide deletions, resulting in frameshift mutations, were detected in a number of genes, with the predicted disruption of their encoded proteins, which are known to play an important role in metabolic pathways and antimicrobial resistance. These results reveal the complexity of dissemination and dynamic evolution of the ST239 lineage in China and indicate possible transmission routes. The availability of next generation sequencing technology on a wider scale will further enhance our understanding of the dynamic evolutionary process in the transmission and spread of globally disseminated multidrug-resistant MRSA.

## Methods

### Bacterial isolates

Four representative ST239 HA-MRSA isolates from bacteremic patients in Hong Kong and Beijing hospitals were selected for whole-genome sequencing. The Hong Kong strains (HK1997 and HK2007) were isolated in 1997 and 2007 at the Dept of Microbiology, Prince of Wales Hospital, Hong Kong, and were representative of strains of indistinguishable PFGE types from a longitudinal surveillance of MRSA strains in Hong Kong in the 1990s to 2007 [4–6]. The Beijing isolates (BJ2002 and BJ2007) were representative of ST239 isolates from bacteremic patients in Beijing in 2002 and 2007, and were strains from the MRSA collection (of Dr H Wang and Dr H Chen) at the Peking Union Medical College Hospital, Beijing. Three complete genomes were used as reference, namely: TW20 [GenBank:FN433596] [19], T0131 [GenBank:CP002643] [11], and JKD6008 [GenBank:CP002120] [12]. For the phylogenetic analyses, sequence data from another 22 global ST239 genomes (collected between 1982 and 2010) were downloaded from the public databases, NCBI Short Read Archive and Whole Genome Shotgun. The downloaded reads data were mapped to TW20 (using software Geneious 6.1.4, Biomatters Ltd., Auckland, New Zealand) and the downloaded contig data were ordered using TW20 as reference. These strains were 16K [GenBank:BABZ00000000], Bmb9393 [GenBank:CP005288], CN79 [GenBank:ANCJ00000000], PPUKM-332-2009 [GenBank:AMRC00000000], PPUKM-775-2009 [GenBank:AMRE00000000], BAA-39 [GenBank:AEEK00000000], MRGR3 [GenBank:AHZL00000000], JKD6009 [GenBank:ABSA00000000], Z172[GenBank:CP006838] [17, 18, 20–22] and ANS46, R35, TUR1, TUR9, TUR27, HU25, HGSA9, FFP103, GRE18, S2, CHI61, S40, DEN907 [SRA: ERA000102] [9, 10]. The ST8 MRSA genome USA300 FPR3757 [GenBank:CP000255], was used as an outgroup to root the ST239 phylogeny [23]. This genome was aligned with all 29 ST239 strains using the software package Mauve (version 2.3.1), and the SNPs exported were used for phylogenetic analyses. As strains were based on MRSA collections from previous epidemiological studies, no clinical data were obtained nor further ethical submission made.

### Molecular typing

The MLST typing was conducted in accordance with the protocol suggested on the MLST website, using seven housekeeping genes (http://saureus.beta.mlst.net/) [24]. Staphylococcus protein A (spa) typing was carried out by sequencing the PCR product of the spa gene, as described, and the spa type was confirmed by using the public spa-type database and Ridom SpaServer (http://spa.ridom.de/; http://tools.egenomics.com/) [25]. SCCmec typing was conducted by multiplex PCR with previously reported primers [26].

### Genome sequencing, assembly and annotation

DNA from each MRSA isolate (1 μg for each sample) was extracted with Wizard Genomic DNA Purification Kit (Promega, Madison, USA), following the manufacturer’s instructions. Whole-genome sequencing was performed using the HiSeq sequencer (Illumina); unique index-tagged libraries were created for each sample to generate 90 bp paired-end reads. These libraries gave more than 400X coverage (sequencing depth) for each strain on average. *De novo* assembly of the sequencing reads was performed using the Velvet (version 1.2.10) software package [27], coupled with the Velvet Optimiser (version 2.2.4) to select the best kmer length for assembly [28]. The assembly statistics of contigs for each genome are provided in Table 2. The scaffold order of the contigs for each genome was determined by mapping to their closely related complete reference genomes (TW20, T0131, and JKD6008), using Mauve (version 2.3.1) [29] and Contiguator 2 software [30]. Assembled contigs were submitted to the RAST server (http://rast.nmpdr.org/) [31] for genome annotation, and manually inspected using Geneious (version 6.1.4) software (Biomatters Ltd., New Zealand).

### Maximum likelihood phylogenetic tree

All genomes of the ST239 representative strains were aligned using the progressive Mauve method with default parameters, and core genome SNPs were retrieved from aligned regions excluding gapped ambiguous columns. The non-core regions were assigned as all sequences that were not present in all 29 ST239 isolates. SNPs were filtered to remove those that were in non-core regions, gaps, and those that included ambiguity codes, and they were finally converted into the phylip format using a PERL script. Maximum likelihood phylogenetic analysis based on core genome SNPs of the ST239 isolates was performed using the RAxML BlackBox [32]. The default CAT model was used, and the ST8 reference genome USA300 FPR3757 [GenBank: CP000255] was included as an outgroup to root the ST239 phylogeny. Supports for nodes were assessed using 100 rapid bootstrap inferences and thereafter by a thorough maximum likelihood search. All free model parameters were estimated by RAxML and likelihood of the final tree was evaluated and optimized under GAMMA.

### Mobile genetic element detection and whole genome alignment

The prophage regions were identified by PHAST (http://phast.wishartlab.com) [33], and the MGE regions in each genome were analyzed by IslandViewer [34]. Whole-genome alignment was performed using Geneious software (Biomatters Ltd., New Zealand) and Mauve (version. 2.3.1) [29] to examine the alignment and distribution of prophage and MGE regions in the genomes.

### Gene ortholog analysis

Predicted genes and their translated protein sequences of the four HK and BJ genomes were compared to those of TW20 and T0131, and clustered into ortholog groups using OrthoMCL software [35]. All versus all BLASTP was performed with the default parameter set (an e-value cut-off of 1 × 10^-5^, a percent match cut-off of 50%, and an inflation value of 1.5). Common and unique orthologous groups identified among the genomes were analyzed using a Venn diagram [36].

### Availability of supporting data

The draft genome sequences of CUHK_HK1997, CUHK_HK2007, CUHK_BJ2002, and CUHK_ BJ2007 have been deposited in the DDBJ/EMBL/GenBank with the accession numbers AZJQ00000000, AZMZ00000000, AZMY00000000, and AZMX00000000, respectively. The versions described in this article are the first versions: AZJQ01000000, AZMZ01000000, AZMY01000000, and AZMX01000000.

Phylogenetic tree data is available in the Dryad Digital Repository (http://datadryad.org/), with the following identifier: http://doi.org/10.5061/dryad.12773.

## Electronic supplementary material

Additional file 1: Figures S1: The structure of pathogenicity island SaPI1 in Beijing Cluster and HongKong Cluster strains. (PDF 193 KB)

Additional file 2: Figures S2: The 18 kb insertion structure in Beijing Cluster strains. (PDF 121 KB)

Additional file 3: Figures S3: The 47 kb phiNM1-like prophage structure in BJ02 strain. (PDF 388 KB)

Additional file 4: Tables S1: The four common functional orthologous gene groups of Beijing cluster strains, which not located on GIs. (XLSX 12 KB)

## Abbreviations

BJ: Beijing
CDS: Coding sequences
GIs: Genomic islands
HA-MRSA: Hospital-associated methicillin-resistant *Staphylococcus aureus*
HK: Hong Kong
MGE: Mobile genetic elements
MRSA: Methicillin-resistant *Staphylococcus aureus*
OGG: Orthologous gene group
SNP: Single nucleotide polymorphism
Spa: Staphylococcus protein A.
