# Supplementary figures and images for: Comparative genomics of methicillin-resistant Staphylococcus aureus ST239: distinct geographical variants in Beijing and Hong Kong
Source: BMC Genomics. 2014 Jun 26;15(1):529. doi: 10.1186/1471-2164-15-529 (PMC4085340; doi:10.1186/1471-2164-15-529)

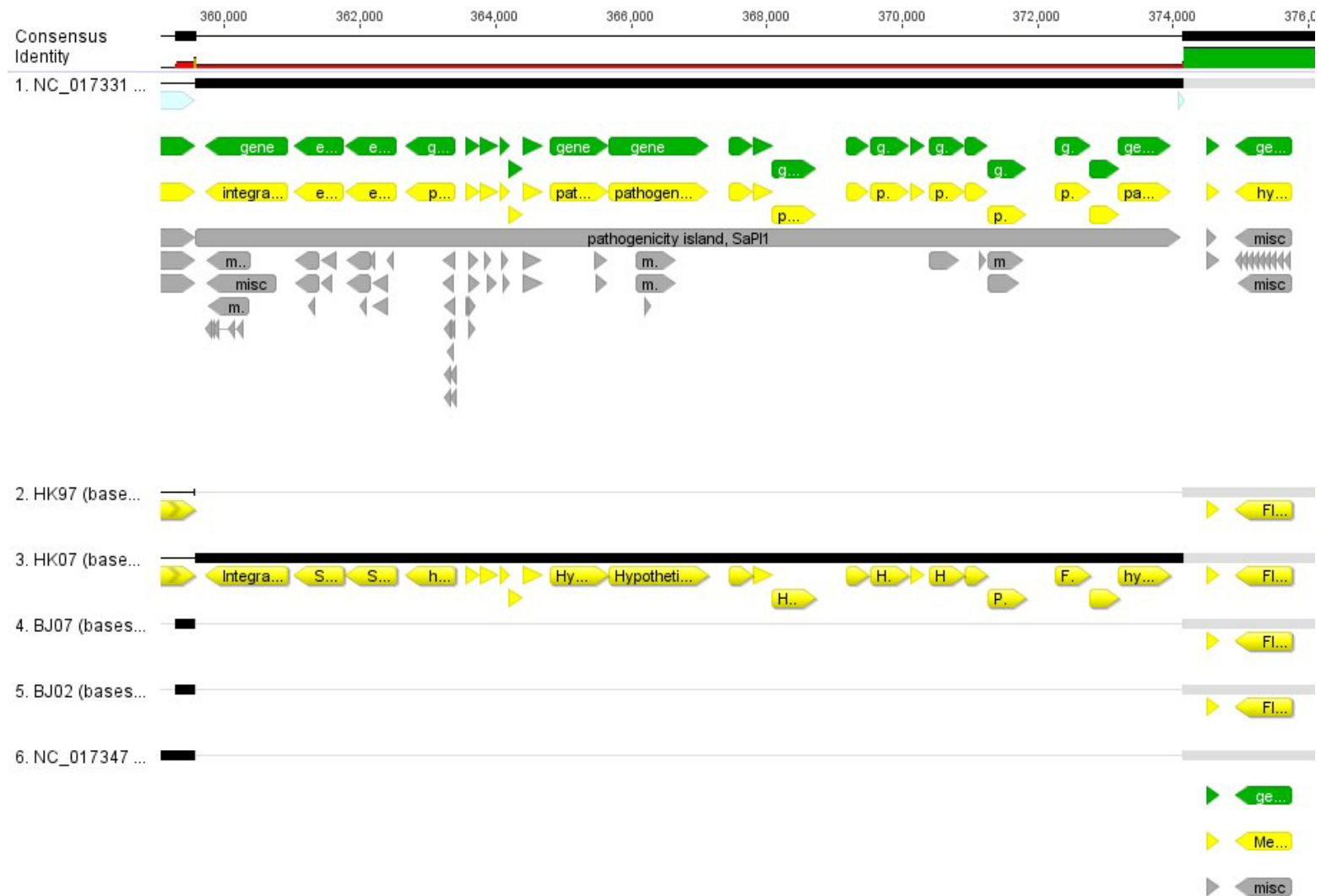

Supplement: Supplementary file 1 — Additional file 1: Figures S1: The structure of pathogenicity island SaPI1 in Beijing Cluster and HongKong Cluster strains. (PDF 193 KB) [file 12864_2014_6203_MOESM1_ESM.pdf]

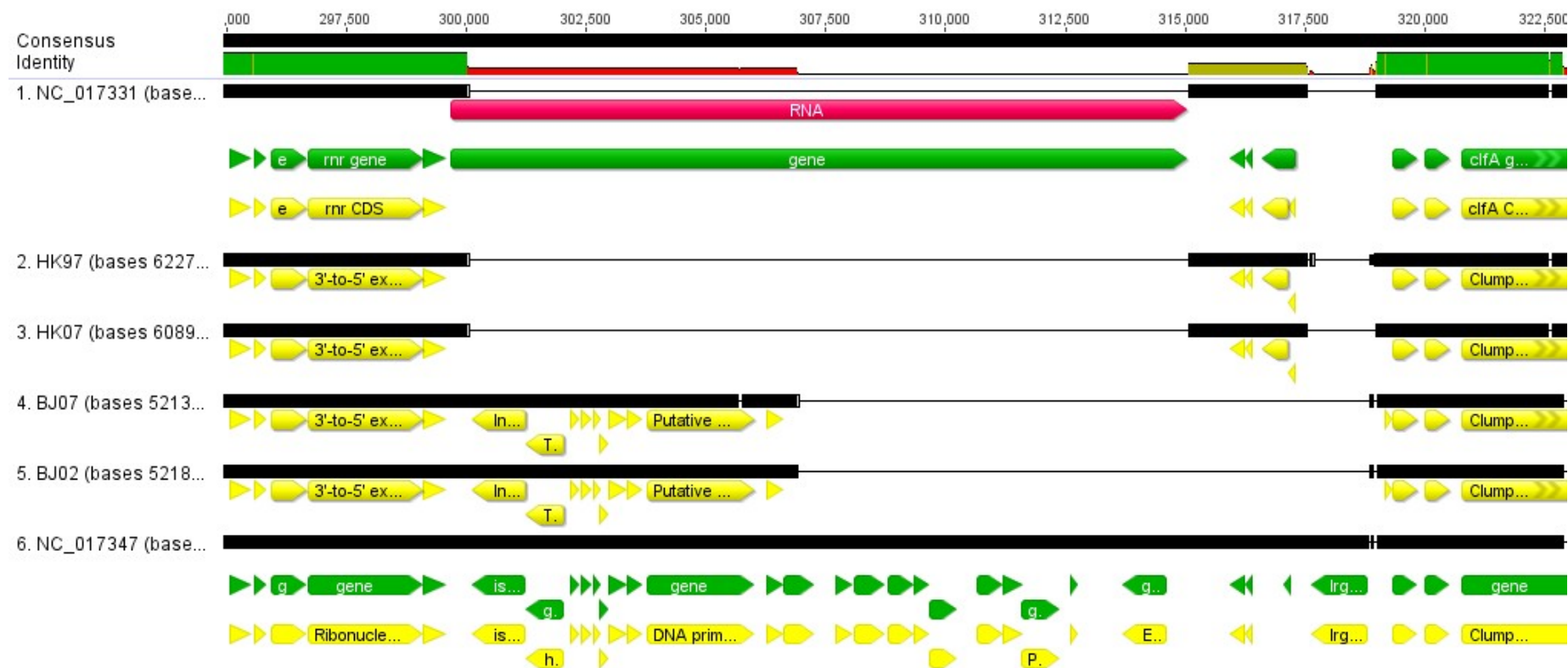

Supplement: Supplementary file 2 — Additional file 2: Figures S2: The 18 kb insertion structure in Beijing Cluster strains. (PDF 121 KB) [file 12864_2014_6203_MOESM2_ESM.pdf]
