# Supplementary material for: Comparative genomics of methicillin-resistant Staphylococcus aureus ST239: distinct geographical variants in Beijing and Hong Kong
Source: BMC Genomics. 2014 Jun 26;15(1):529. doi: 10.1186/1471-2164-15-529 (PMC4085340; doi:10.1186/1471-2164-15-529)
Supplement: Supplementary file 3 — Additional file 3: Figures S3: The 47 kb phiNM1-like prophage structure in BJ02 strain. (PDF 388 KB) [file 12864_2014_6203_MOESM3_ESM.pdf]

Prophage region: 2
Number of CDS: 67
Location: from 1904132 to 1951671 (47540 bps)
Predicted status: intact prophage
GC content: 34.05%

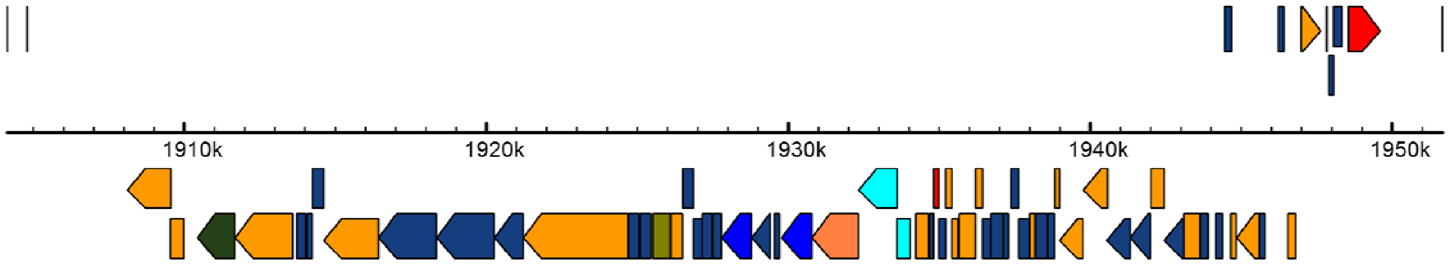

Identified CDS types:

	1	Lysis		2	Terminase		3	Portal
	4	Protease		5	Coat		6	Tail shaft
	7	Attachment site		8	Intergrase		9	Other phage-like protein
	10	Hypothetical protein		11	Other		12	Transposase
	13	Tail fiber		14	Plate		15	tRNA
